# The Phenotypic Spectrum of 47 Czech Patients with Single, Large-Scale Mitochondrial DNA Deletions

**DOI:** 10.3390/brainsci10110766

**Published:** 2020-10-22

**Authors:** Nicole Anteneová, Silvie Kelifová, Hana Kolářová, Alžběta Vondráčková, Iveta Tóthová, Petra Lišková, Martin Magner, Josef Zámečník, Hana Hansíková, Jiří Zeman, Markéta Tesařová, Tomáš Honzík

**Affiliations:** 1 Department of Paediatrics and Inherited Metabolic Disorders, First Faculty of Medicine, Charles University and General University Hospital, Ke Karlovu 2, 128 08 Prague 2, Czech Republic; nicole.anteneova@vfn.cz (N.A.); silvie.kelifova@vfn.cz (S.K.); hana.kolarova@vfn.cz (H.K.); alzbeta.vondrackova@lf1.cuni.cz (A.V.); iveta.tothova@vfn.cz (I.T.); petra.liskova@lf1.cuni.cz (P.L.); martin.magner@vfn.cz (M.M.); hana.hansikova@vfn.cz (H.H.); jzem@lf1.cuni.cz (J.Z.); tomas.honzik@vfn.cz (T.H.); 2 Department of Ophthalmology, First Faculty of Medicine, Charles University and General University Hospital, U Nemocnice 2, 128 08 Prague 2, Czech Republic; 3 Department of Paediatrics, First Faculty of Medicine, Charles University and Thomayer Hospital, Vídeňská 800, 140 59 Prague 4, Czech Republic; 4 Department of Pathology and Molecular Medicine, Second Faculty of Medicine, Charles University and Motol University Hospital, V Úvalu 84, 150 06 Prague 5, Czech Republic; josef.zamecnik@lfmotol.cuni.cz

**Keywords:** Kearns-Sayre Syndrome (KSS) Spectrum, Progressive External Ophthalmoplegia (PEO), Pearson Syndrome, mtDNA, Single, Large-Scale Mitochondrial DNA Deletions (SLSMD)

## Abstract

Background: In this retrospective study, we analysed clinical, biochemical and molecular genetic data of 47 Czech patients with Single, Large-Scale Mitochondrial DNA Deletions (SLSMD). Methods: The diagnosis was based on the long-range PCR (LX-PCR) screening of mtDNA isolated from muscle biopsy in 15 patients, and from the buccal swab, urinary epithelial cells and blood in 32 patients. Results: A total of 57% patients manifested before the age of 16. We did not find any significant difference between paediatric and adult manifestation in either the proportion of patients that would develop extraocular symptoms, or the timespan of its progression. The survival rate in patients with Pearson Syndrome reached 60%. Altogether, five patients manifested with *atypical* phenotype not fulfilling the latest criteria for SLSMD. No correlation was found between the disease severity and all heteroplasmy levels, lengths of the deletion and respiratory chain activities in muscle. Conclusions: Paediatric manifestation of Progressive External Ophthalmoplegia (PEO) is not associated with a higher risk of multisystemic involvement. Contrary to PEO and Kearns-Sayre Syndrome Spectrum, Pearson Syndrome still contributes to a significant childhood mortality. SLSMD should be considered even in cases with *atypical* presentation. To successfully identify carriers of SLSMD, a repeated combined analysis of buccal swab and urinary epithelial cells is needed.

## 1. Introduction

Single, Large-Scale Mitochondrial DNA Deletions (SLSMD) are common causes of multisystem mitochondrial diseases typically associated with Kearns-Sayre Syndrome (KSS) Spectrum, Progressive External Ophthalmoplegia (PEO) and Pearson Syndrome [1,2]. SLSMD occur sporadically *de novo*, in most cases during the process of mitochondrial DNA (mtDNA) replication [3], with 4% risk of transmission from mother to offspring [4]. However, a mutation in the nuclear gene *SSBP1* (Single-Stranded DNA-Binding Protein 1) was recently described, causing SLSMD with clinical symptomatology of Pearson Syndrome, KSS and Leigh syndrome [5]. Although, most common SLSMD is about 5 kb (4977 bp; m.8470_13446del4977), there is a significant variation of both location and size (1.3–10 kb) [3,6]. SLSMD are heteroplasmic and can be detected in various human tissues [6,7]. The exact prevalence is unknown, but it has been estimated at 1.5: 100,000 [8].

Originally, PEO was characterized by slowly progressive bilateral eyelid ptosis and ophthalmoparesis, that was sometimes associated with dysphagia, proximal limb weakness and/or exercise intolerance also known as *PEO plus* [9]. Classical criteria of KSS formulated in 1983 were defined by typical triad of PEO, pigmentary retinopathy, and onset of symptoms before the age of 20 [10]. Pearson Syndrome was characterised as sideroblastic anaemia and variable involvement of other blood cell lines, together with exocrine pancreatic dysfunction [11]. These former definitions did neither fully cover clinical phenotype of SLSMD syndromes nor did classify all patients with confirmed SLSMD. Therefore, new diagnostic criteria determined in 2015 by the group of Mancuso reformulated classical criteria [2]. These extend classical definition of KSS and introduce the so called “KSS Spectrum”, that better reflect multisystem involvement and take into account possible late-onset phenotypes [2]. Pearson Syndrome is newly defined as a paediatric sideroblastic/megakaryocytic anaemia that may or may not evolve into KSS Spectrum; pancreatic insufficiency should be taken as additional symptom variably present [2,12]. PEO is characterised as ptosis and/or ophthalmoparesis due to SLSMD that neither fulfil KSS Spectrum criteria nor criteria for Pearson Syndrome [2].

Although, the exactness of the clinical criteria is often a big help for the clinician, symptomatology may sometimes greatly differ which may cause considerable diagnostic difficulties, delaying the communication of correct diagnosis and initiation of proper care. Herein, we present the clinical, biochemical and molecular data of Czech patients diagnosed with SLSMD. The aim of this retrospective cohort study was to describe natural course of the disease with a focus on the progression from PEO to KSS Spectrum. In addition, our experience with the reliability of available diagnostic methods is provided, in order to optimize the management of these underdiagnosed multisystem disorders.

## 2. Materials and Methods

### 2.1. Group Characterization

Between 1992 and 2020, 47 Czech patients with SLSMD detected at least in one tissue were diagnosed at our institution. Both clinical and laboratory data of a total of 23 males and 24 females from 47 unrelated families were collected and analysed in this retrospective cohort. The group description is summarized in Table 1. Patients were phenotyped using recently proposed KSS Spectrum criteria [2]. Fully expressed disease was found in 20/47 patients (42.5%, 9 females, 11 males); five patients (10.6%, 3 females, 2 males) were diagnosed as Pearson Syndrome and 17 patients (36.2%, 10 females, 7 males) were classified as PEO. Five patients (10.6%, 2 females, 3 males) with SLSMD did not fulfil the phenotypical criteria of SLSMD syndrome and were, therefore, classified as “*atypical*” and were included in the primary cohort. Informed consent has been obtained from the patients or their legal representatives prior to the analyses. The Pearson Syndrome patients were previously published but we decided to include them in the present study to provide full spectrum of phenotypes associated with SLSMD [13].

### 2.2. Molecular Genetic Analyses

Long-range PCR (LX-PCR) amplification of mtDNA has been used as a screening test to detect deletions of mtDNA molecules in patient samples since 2004. The Southern blot of muscle DNA samples was utilized for SLSMD diagnosis in patients recruited before 2004. The initial LX-PCR protocol amplified mtDNA region m.161–m.145 using TAKARA LA Taq Polymerase (TaKaRa Bio, Kusacu, Japan). In 2008 the protocol was changed and primers (F5459-5493 and R735-701) published by Amati-Bonneau et al. and LA Hot Start Master Mix (TopBio, Vestec, Czech Republic) were employed [14]. Samples with detected SLSMD by Southern blot were retested by LX-PCR. In total, 265 DNA samples were tested by LX-PCR: more than one tissue was tested in each patient; easily available tissues were tested repeatedly in majority of patients. The tissues tested include blood, urinary epithelial cells, buccal swab cells, muscle biopsy, bone marrow, autoptic tissues, hair follicles. Total genomic DNA was isolated from collected samples prior to LX-PCR. For a characterization of deletion break-points and an assessment of its heteroplasmy level, SeqCap EZ Design: Mitochondrial Genome Design (Roche NimbleGen, Pleasonton, CA, USA) enrichment kit was used for preparation of sequencing library, followed by analysis on MiSeq (Illumina, San Diego, CA, USA) system using MiSeq Reagent Sequencing kit v3. Eighty-five DNA samples were available for precise characterization of SLSMD (35 muscle biopsies, 28 blood samples, 2 buccal swabs, 2 bone marrow samples, 18 autoptic tissues; more than one tissue was tested in each patient, 2 independent DNA isolations were available from 3 muscle biopsies). MtDNA deletion break-points were unequivocally characterized in 29 samples.

Exons of the *SSBP1* gene (NM_001256510) were analysed by Sanger sequencing, exome sequencing proceeded as described previously [15].

## 3. Results

### 3.1. Patients Characterization

#### 3.1.1. Disease Onset

The age of onset varied in our cohort from early neonatal period to 66 years of age. Twenty-seven (57%) patients from the cohort were diagnosed before the age of 16. The most common presenting symptoms were isolated ptosis (14/42, 33%), anaemia (4/42, 10%), failure to thrive (3/42, 7%) and isolated ophthalmoparesis (3/42, 7%).

##### PEO

In PEO patients the mean age at onset was 20.5 ± 12 years (range 6–41 years); in 9 patients (53%), the first symptom developed before the age of 16. The most common presenting symptom was eyelid ptosis (7 patients, 41%); three patients (18%) manifested as isolated ophthalmoplegia, another three as a combination of both, and in two patients (12%) isolated diplopia was present. In addition, one patient had both headache and diplopia as a first symptom and another one had a combination of ptosis and dysphagia.

##### KSS Spectrum

In KSS Spectrum group the mean age at onset was 18.2 ± 12.2 years (range was neonatal to 45 years). The first symptoms developed before the age of 16 in 11 patients (55%). The most common first symptom was symmetric bilateral ptosis (7 patients, 35%). To other initial symptoms belonged ophthalmoparesis, accompanied with ptosis, and ophthalmoparesis together with skeletal muscle weakness (both in two patients). Interestingly, two paediatric patients were firstly investigated for failure to thrive and within a few years they developed ptosis and neurological symptomatology. In one child, a psychomotor delay was initially noted, but a multisystemic involvement with pancreatitis, short stature, nephropathy, pigmentary retinopathy, ptosis and hearing impairment developed before he reached the age of 18.

##### Pearson Syndrome

All patients with Pearson Syndrome manifested before 1 year of age. Presenting complaint in 4 patients was transfusion dependent sideroblastic anaemia. The fifth male patient manifested by failure to thrive and required only one transfusion due to macrocytic sideroblastic anaemia at the age of five. He further developed symptoms corelating with KSS Spectrum.

#### 3.1.2. Clinical Symptoms and Disease Progression

Clinical symptoms of all patients are summarized in Table 2.

##### PEO

Both ptosis and ophthalmoparesis developed in all 17 patients with PEO (100%). Apart from ophthalmological symptoms, muscular involvement was present collectively in 8 cases. A total of 4/8 individuals suffered from exercise intolerance; 4/8 had swallowing difficulties and 3/8 had myalgia. Nine of our patients (53%) developed first symptoms during childhood at the age of 11 ± 4 years. The current mean age of our patients with PEO is 43 ± 14 years. Duration of follow-up of our patients with PEO phenotype was 14.5 ± 11.8 years; the duration of follow-up of paediatrics patients did not differ for the adult group. During our follow-up, none of our PEO patients have progressed to KSS Spectrum. There are no deceased patients in this group.

##### KSS Spectrum

Eyelid ptosis developed in all patients classified as KSS Spectrum; majority of them suffered from ophthalmoparesis (80%) as well. From a total of 28 patients firstly manifesting as PEO, 9 remained with only ocular manifestation during a follow up of 18.7 ± 12 years (average age of onset of patients with isolated PEO was 21.6 ± 11.3 years). However, eight patients developed muscular symptoms, formerly known as *PEO plus* during median of 10 ± 8.3 years and 11 patients developed during median of 11 ± 7.4 years symptoms fulfilling criteria for KSS Spectrum. One patient developed bilateral corneal endothelial failure. The most common extraocular symptom in the whole KSS Spectrum group was exercise intolerance (16/20); 12/20 (60%) patients developed pigmentary retinopathy and 10/20 (50%) hearing impairment. Cerebellar symptomatology was noted in 10 patients (10/20; 50%). Cardiovascular symptoms were present in 10 patients; in 6/20 patients an atrioventricular conduction block was diagnosed with its third-degree block manifesting in five patients. One patient suffered from dilated cardiomyopathy. Two patients (10%) had renal Fanconi Syndrome, other two were diagnosed as having mild cognitive impairment. Six patients with KSS Spectrum (6/20; 30%) died at the mean age of 37 ± 21.3 years (1 female, 5 males), median is 21 ± 5.5 years after the onset of disease. The median age of surviving patients with KSS Spectrum is 30 ± 17 years.

##### Pearson Syndrome

All patients underwent bone marrow aspiration with pathognomonic sideroblastic macrocytic anaemia with vacuolization of bone marrow precursors. Two patients (5%) had microcephaly and other two had primary adrenal insufficiency. Three patients were diagnosed with cognitive impairment. Three patients (60%) had short stature and one of them was temporarily administrated with growth hormone therapy. Four (80%) of them also had failure to thrive. In the surviving patients with Pearson Syndrome, two patients developed neuromuscular symptomatology, one fulfilling criteria of KSS Spectrum. Two patients died before the age of 1 year due to infection with metabolic derangement irresponsive to therapy; both had refractory anaemia and one also had pancreatic insufficiency.

#### 3.1.3. Atypical Phenotype

Patients with multisystem clinical manifestation not fulfilling the SLSMD criteria, with detection of SLSMD are classified as having an *atypical* phenotype. Mean age at onset was 29.7 ± 26.8 years (range 1.5–66 years). Patient 1 was diagnosed before the age of two with psychomotor delay, during his childhood exercise intolerance, moderate cognitive impairment, hypertrophic cardiomyopathy, short stature, and lactate acidosis developed. Patient 2 manifested at 17 years with epilepsy, but within the course of the disease dysarthria, cerebellar symptomatology, and myopathy appeared, he was lost from follow-up and died of unknown cause at 57 years. Patient 3 was diagnosed at 15 years with dysarthria and hypomimia but developed muscle atrophy and dysphagia at age of 32. Two individuals manifested first symptoms during adulthood. Patient 4 presented at 66 years with hearing impairment; during a five-year span, she developed neuromuscular symptoms consisting of myalgia, dysphagia, exercise intolerance, and polyneuropathy. Patient 5′s first complaint was myalgia at age of 49, later followed by diabetes mellitus and exercise intolerance. All patients eventually developed multisystemic manifestation of the disease but none of them suffered from ptosis, or ophthalmoparesis nor sideroblastic anaemia within 25.3 ± 9.5 years of follow-up. Clinical data of patients with *atypical* phenotype are further summarised in Table 3.

### 3.2. Biochemistry

Mild or severe elevations in blood lactate levels were found in 17/34 (50%) patients (maximum 56.3 mmol/L), in 18/32 (56%) patients (maximum 3773 mmol/mol Kr) in urine, and in 3/6 patients (maximum 6.8 mmol/L) in cerebrospinal fluid (CSF). Metabolic tests showed elevated levels of serum alanine (reference value < 500 μmol/L) in 21/38 (55%) patients with maximum 2379 μmol/L (median 612 μmol/L). Three patients (two with Pearson Syndrome, one with KSS Spectrum) had increased excretion of intermediate metabolites of the Krebs cycle—the range (median) were 223–912 μmol/L (267.5 μmol/L) and 27–214 μmol/L (median 113 μmol/L) for 2-oxoglutarate and fumarate, respectively. CSF 5-methyltetrahydrofolate (5-MTHF) was assessed in three patients (two with KSS Spectrum and one with Pearson Syndrome)—in all of them with lower levels (reference value > 40 nmol/L; KSS Spectrum: 0.1–23.7 nmol/L; Pearson Syndrome: 4.5 nmol/L) resulting in folinate supplementation (0.6 mg/kg/day–1 mg/kg/day). Creatine kinase (CK) was detected in 34/47 (72%) of patients, the range is 0.21–70.06 μkat/L (median 2.44 μkat/L). Altogether, 11 patients had at least one pathological value of CK (male reference values: 0–15 years < 2.27 μkat/L, 15–99 years < 5.14 μkat/L; female reference values: 0–15 years < 2.27 μkat/L, 15–99 years < 3.21 μkat/L) within the course of their disease.

### 3.3. Muscle Analysis

Muscle biopsy was performed in 32/47 (68%) patients. Ragged red fibres (RRF) were observed in 13 patients; focal subsarcolemmal accumulation of succinate dehydrogenase (SDH) reaction product was detected in nine patients, and 21 patients had cytochrome *c* oxidase (COX) negative muscle fibres.

Activity of respiratory chain complexes were measured by spectrophotometry in 25 muscle samples (methodology described in Danhelovska et al. [16]). Activities within reference range was found only in 4/25 samples. The 21/25 samples demonstrated decreased activity of at least one respiratory chain complex, however combined deficiencies were prevalent.

### 3.4. Neuroimaging

Brain magnetic resonance imaging (MRI) showed abnormal findings in 15/27 (56%) patients—revealing atrophy or abnormal signals in various areas of the brain (basal ganglia, cortex, periventricular, cerebellar). One patient with Pearson Syndrome had extensive high T2 signal intensity typical for Leigh Syndrome, accompanied by lesions in supratentorial white matter and in atrophic cerebellum. One patient with *atypical* phenotype had T2 high intensity in frontal area of cerebrum and another one with PEO in corpus callosum; three patients had cortical atrophy, one had cerebellar atrophy and another one had periventricular atrophy.

### 3.5. Molecular Genetics

The SLSMD was detected by LX-PCR at least in one DNA sample obtained from every patient. The tested tissues depended on the availability of samples from individual patients. In some patients, the SLSMD was detected in all available tissues. MtDNA deletion break-points were unequivocally characterized in 29 out of 85 samples obtained from 18 patients (38%). The detected length of the deletion was 1835–7827 bp. In our cohort 6/18 (33%) patients harboured the most common deletion about 5 kb long (3/6 patients had the same deletion—4963 bp). The PEO’s group (eight patients) deletion range was 2298–7535 bp (median 4963 bp). The range of the deletions in the KSS Spectrum’s group (eight patients) was 1835–7827 bp (median 4969 bp). The Pearson Syndrome’s group (two patients) deletion was 4963 bp and 5395 bp. In these 18 patients, there was no significant correlation between the age of presentation and the size of the deletion. Heteroplasmy level detected in the PEO group (eight patients) was in the muscle 39–76% (median was 58%). In the KSS Spectrum group heteroplasmy detected in the muscle (7 patients) was 58–91% (median was 66%), in the blood (2 patients) was 56% and 69%.

The SLSMD length remains unchanged, only heteroplasmy levels varies among various tissues of individual patient. Post-mortem examination of the tissues of one patient showed a difference between heteroplasmy in each tissue: the kidney 79.5%, the cerebellum 65%, the cerebrum 84%, the liver 51%, the muscle 91% and the heart 41%. In the Pearson Syndrome group, two patients’ heteroplasmy levels detected in the muscle were 30% and 71% (the same patient’s heteroplasmy level in the bone marrow was 31%); one patient’s tissues were examined post-mortem with findings of heteroplasmy levels in the heart 43% and in the cerebrum 83%.

**Figure 1:**
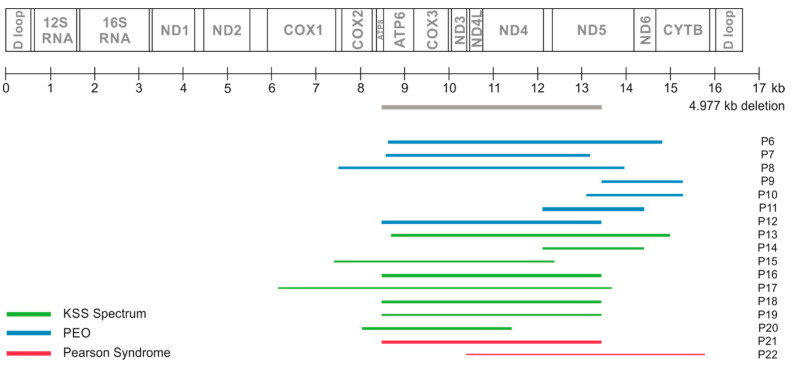
Molecular-genetic data characterizations of SLSMD in muscle of 17 patients. Colour of bars correspond with phenotypic group, thickness of the bars corresponds with heteroplasmy level. The exact lengths and heteroplasmy levels were as follows: P6—6193 bp, 72%; P7—4616 bp, 66%, P8—6457 bb, 58%; P9—1835 bp, 59%; P10—2184 bp, 57%; P11—2298 bp, 91%; P12—4961 bp, 78.5%; P13—6302 bp, 68%; P14—2298 bp, 56%; P15—4978 bp, 46%; P16—4963 bp, 76%; P17—7535 bp, 39%; P18—4963 bp, 64%; P19—4963 bp, 46%; P20—3387 bp, 60%; P21—4963 bp, 71%; P22—5395 bp, 30%. For clarity, the 4.977 kb mtDNA deletion (e.g., common deletion) is included at heteroplasmy 100% (grey).

The SLSMD characterization of 17 patients (tested from muscle) are summarized in Figure 1.

In the majority of patients (26/47), the diagnosis of SLSMD was determined due to analysis of muscle biopsy (it was the first tissue available for the analysis). In six patients, muscle biopsy was inevitable to confirm the SLMSD diagnosis since previously tested tissues (buccal swab, urinary epithelial cells, and/or blood) were negative (three patients) or inconclusive (three patients). The diagnosis of SLSMD in the remaining 15 patients was based on the LX-PCR screening of mtDNA isolated from buccal swab, urinary epithelial cells or blood.

In 33 patients, blood (46 samples), urinary epithelial cells (28 samples), and buccal swab (40 samples) were available for the SLSMD testing at least once. The SLSMD was detected every time in blood of 10 (out of 30) patients (33%), buccal swab of 18 (out of 27) patients (66%), and in urinary epithelial cells of 14 (out of 22) patients (63%).

Patients with *atypical* SLSMD phenotype mutation in nuclear gene *SSBP1* were excluded. Exome sequencing in P3 did not provide any variant which could be responsible for the patient phenotype.

## 4. Discussion

Herein, we report the phenotypical and molecular data of 47 Czech patients with SLSMD. Despite the fact that SLSMD are nowadays considered as having the age of onset occurring anytime from infancy to adulthood, more than half of our patients were diagnosed before the age of 16 [2,17,18], making it still a childhood disease with a need of its consideration in any progressive multisystemic disease in a paediatric patient. Although the female to male ratio was a little higher in two previously published large cohort studies (Mancuso et al.; Yamashita et al.), we did not find any sex predilection in our study [2,17].

The major phenotype in our group was KSS Spectrum (42.5%), followed by PEO (36.2%) and Pearson Syndrome (10.6%). This is not in accordance with the two larger studies, where patients with PEO were the most represented (65–70%) and patients with Pearson Syndrome made up less than 5% [2,17]. Since another five patients (10.6%) had *atypical*, but also multisystemic involvement, it is obvious, that the majority of our patients were on the more severe part of the disease spectrum. This may be caused by the fact that only severely affected patients are eventually referred to our institution, and mildly affected PEO patients often remain under- or misdiagnosed. The need for a systematized screening in all neuromuscular units is therefore of utmost importance.

Half of patients with PEO were diagnosed during childhood. It would be plausible to suggest that a childhood manifestation of PEO would lead to more severe phenotype with higher probability to progression as in other mitochondrial disorders, such as MELAS Syndrome [19]. However, we did not find any significant difference between paediatric and adult patients in either the proportion of individuals that would develop additional muscular or KSS Spectrum symptoms, or the timespan of this progression. Two thirds of our patients manifested at the beginning by isolated ocular symptoms but later developed systemic involvement (10 ± 7.4 years). The longest follow-up of our patient with isolated PEO was 38 years, but meanwhile other patient had monosymptomatic PEO for as long as 29 years yet still developed complete KSS Spectrum. It seems that the clinical course of the disease is not predictable based on the age of onset or the length of monosymptomatic manifestation. Long-term follow-up is therefore highly recommended.

Patients diagnosed with PEO usually suffer from symmetric ptosis and ophthalmoparesis [20]. However, in some cases, ptosis may precede ophthalmoparesis for years, while ophthalmoparesis without ptosis is rare [21]. All of our patients eventually developed fully expressed PEO with both ptosis and ophthalmoparesis. Nevertheless, 23% of patients had isolated ophthalmoparesis for as long as 18 years, with only nine simultaneously reporting diplopia. While in ptosis the recognition is often soon, the diagnosis could be otherwise delayed for years or even missed in patients with isolated ophthalmoparesis, especially in the absence of the physical examination to check out the eye movements.

Altogether, 11/20 (55%) patients with KSS Spectrum manifested before the age of 16. Interestingly, half of the paediatric patients were at the beginning thoroughly investigated for growth impairment, failure to thrive, tremor, psychomotor delay, or hearing impairment. Nevertheless, in a median of 5.5 ± 5.3 years developed other symptoms fulfilling the definition of KSS Spectrum. Mortality rate in the group of patients with KSS Spectrum was slightly higher (6/20, 30%) when compared to other studies, where it reached 11–20% [2,18,22]. This is in line with the fact, that a larger proportion of our patients had severe phenotype corresponding to KSS Spectrum or Pearson syndrome, and hence, were more prone to develop life-threating complications.

In our cohort, five patients were diagnosed with Pearson Syndrome in infancy, but only one of them had a history of pancreatic insufficiency as in the classically described condition [11]. This is in line with recent studies, where only 12–36% of patients with Pearson Syndrome manifest with exocrine pancreatic dysfunction [2,22,23,24], and it brings further support for its exclusion of the diagnostic criteria. Although transfusion-dependent sideroblastic anaemia is a key finding in Pearson Syndrome [11], we report a patient with failure to thrive as a leading complaint and only mild sideroblastic anaemia with sideroblasts in bone marrow requiring only single transfusion at the age of five. Similarly, as in KSS, incomplete phenotypes seem to be also common in patients with previously rather strictly defined Pearson Syndrome. The progression into KSS Spectrum in our group (20%) is in agreement with published studies (18–27%) [22,24]. It is of note that two of our patients with Pearson Syndrome manifested with adrenal insufficiency with severe adrenal crisis and as one of the presenting symptoms in a 4-year-old boy. This is rather underreported finding when it comes to Pearson Syndrome, since it was described only in two patients so far [25]. Contrary to our patients with PEO and KSS Spectrum, where five-year survival reached 100%, in patients with Pearson Syndrome it was only 60%. Similarly to our observation, five year survival was higher in patients with KSS Spectrum or PEO when compared to patients with Pearson Syndrome [22]. An Italian retrospective cohort study showed survival around 20% after median follow-up period of 5.7 years [25]. Compared to PEO and KSS Spectrum, Pearson Syndrome still contributes to a significant morbidity and reduction in life expectancy.

Five of our patients with confirmed SLSMD did not fulfil the new criteria for any of the three clinical syndromes as defined by Mancuso et al. [2], and were therefore classified as having *atypical* phenotype. Interestingly, two of these patients manifested before the age of 16 with rather non-specific symptoms (psychomotor delay and dysarthria with hypomimia, respectively). The diagnosis of SLSMD in this group of patients was therefore established rather late (18.6 ± 13.2 years after the disease onset). Individual cases of patients with non-Pearson SLSMD but without the cardinal features of ptosis, ophthalmoparesis, or sideroblastic anaemia, have been occasionally described in the literature, all with onset in childhood [17,22]. It seems that patients with paediatric onset of SLSMD may present with *atypical* or non-specific symptoms of the disease, which may cause considerable diagnostic difficulties.

Impaired cognitive function was diagnosed in six of our patients with SLSMD (13%)—two patients had KSS Spectrum, three had Pearson Syndrome and one was classified as *atypical* phenotype. This is rather a high number when compared to other studies [2,26]. Moreover, in the study by Bosbach et al., neuropsychological testing of patients with KSS Spectrum and PEO did not reveal general intellectual deterioration, but only specific cognitive deficits (visual construction, attention, and flexibility) [26]. However, all our patients had an overall deterioration of cognitive skills ranging from mild mental insufficiency to severe deterioration of intellectual abilities within the range of dementia. Routine psychological evaluation should be included in the diagnostic criteria, especially due to its influence on the development of physical disability.

The majority of our patients underwent standard metabolic biochemistry examination; CSF was examined only in a fraction of patients as it is no longer required for establishing the diagnosis of KSS Spectrum [10]. Nevertheless, there was a significant deficiency of cerebral folate (CFD) in all three patients undergoing lumbar puncture. This is in line with previous observations of CFD in patients phenotypically classified as KSS Spectrum [27,28,29,30,31,32]. The underlying pathophysiological mechanism seems to be an energetic defect on the level of choroid plexus cells due to accumulated mutated mtDNA leading to impaired folate transport [33]. However, only scarce data about folinic acid supplementation and its positive effect in patients with KSS were published so far [29,30,34]. Based on our findings, we assume that CSF 5-MTHF monitoring should be addressed especially in severely affected patients with KSS Spectrum and Pearson Syndrome, in order to initiate the early supplementation with folinic acid. Nevertheless, detailed data on the biochemical changes of 5-MTHF metabolism within the course of the disease are recommended for future research.

The correlation between the size of mitochondrial DNA deletion, tissue heteroplasmy and phenotypic manifestations has not been cleared up yet. We assumed that patients with more severe phenotypic expression (Pearson Syndrome or KSS Spectrum) would have a longer deletion than patients with PEO. Nevertheless, we have not found any correlation between the heteroplasmy levels, the size and location of the deletion, and the severity of the clinical manifestation or the disease onset. However, it is of note that only two patients with Pearson Syndrome have been completely genetically examined. Similar to our observations, the location of the deletion itself did not differ in patients with KSS and PEO, nor did it correlate with the onset of symptom [35]. On the other hand, conclusions of a larger cohort study of Mancuso et al. involving 225 patients was that the length of the deletion correlated with more severe phenotypic expression and the rate of heteroplasmy was higher in patients with earlier onset, but heteroplasmy level from muscle did not correlate with the severity of the clinical picture [2]. Similarly, the size of the deletion, in contrast to the degree of heteroplasmy in muscle, correlated with a more severe phenotypic manifestation, but also with an earlier manifestation of the disease [17]. In addition, a direct relationship between both the size and the location of mtDNA deletion, its heteroplasmy and phenotypic (activities of respiratory chain complexes) manifestations was recently demonstrated on the single muscle-fibre level [6] and the pathogenic mechanisms associated with SLSMD was clarified. We hypothesize that severity of the clinical phenotype including early age of onset might be determined by the distribution and accumulation of deleted mtDNA molecules among cells and tissues during embryonic and foetal development. Further carefully designed studies on a single cell lines level with a more homogenous distribution of all three major phenotypes would be necessary to demonstrate the relationship between the mtDNA deletion and the disease manifestation.

All deletion mutations detected in our patients were *de novo* with no confirmed transfer of SLSMD to other family members, which is not in accordance to 4% risk of transmission to offspring mentioned in literature [4]. A total of eight patients (33%) harboured the common 5 kb deletion with variable presentation among the SLSMD spectrum. In the studies of Mancuso et al. and in Broomfield et al., 41% and 73% of patients harboured the common deletion (about 5 kb), respectively [2,22]. Sporadic events during mtDNA replication processes have long been thought to contribute exclusively to SLSMD. Nevertheless, a *de novo* nuclear mutation in *SSBP1*, was recently discovered to cause SLSMD in patients with KSS Spectrum or Pearson Syndrome phenotypes [5]. In order to address the eventual contribution of other variants, we performed exome sequencing in one patient and *SSBP1* gene sequencing in all our patients with *atypical* phenotype, but none another relevant pathogenic variant was found.

As the functional examination of muscle tissue in the diagnosis of mitochondrial diseases is currently being abandoned, the diagnosis of extensive deletions of mtDNA is moving towards analysis of easily and non-invasively accessible tissues (buccal swab, urine or peripheral blood). Moreover, functional and histochemical studies in muscle biopsies in our cohort neither provided any finding specific to SLSMD, nor was it useful for disease course prediction. The shift from the invasive muscle biopsy examination to screening of easily accessible tissue has been clearly demonstrated in our cohort since the diagnosis of SLSMD in 15 our patients was possible only based on analyses of urinary sediment and/or buccal swab. Recently, Varnaugh et al. also confirmed that urine can be used to screen patients suspected clinically of having a single mtDNA deletion [36]. We suggest that using a combination of buccal swabs and urinary epithelial cells may be rather effective diagnostic tool for mtDNA deletion detection. This non-invasive screening should be considered especially in children with non- specific beginning of disease. However, the mtDNA deletions were detected in every buccal swab and urine epithelial cells collected from approx. 64% of our patients. Likewise, repeated testing of these tissues might be necessary in order to establish the correct diagnosis. Only in rare, selected cases, where the latter was not confirmatory, a muscle biopsy examination must be performed. In line with this statement is the fact, that 3/5 of our patients with atypical phenotype underwent muscle biopsy.

Given the estimated prevalence of SLSMD at 1.5: 100,000, these diseases seem to be highly under-/misdiagnosed in the Czech Republic [8]. This is especially true for individuals with milder phenotype manifesting as isolated PEO. Non-invasive screening of easily accessible tissues, such as urinary sediment or buccal swab would represent an effective diagnostic tool that should be introduced across all disciplines, but especially to neuromuscular units.

## 5. Conclusions

The majority of patients with SLSMD manifest during childhood age. These severe mitochondrial diseases should be considered especially when multisystemic phenotype is present, even in the absence of its cardinal features, including ptosis, ophthalmoparesis and myopathy. Children who display early-onset PEO are at a similar risk for developing KSS Spectrum, as these present with later manifestations. Contrarily to other mitochondrial diseases, the age of onset is therefore not helpful in predicting the disease severity.

The newly described phenotype of *atypical* presentation of SLSMD, as confirmed by additional genetic examination, expands the clinical spectrum of these disorders. The wide-span age of onset and non-specific symptoms in the paediatric population bring considerable diagnostic difficulties and emphasize the need for a relevant diagnostic marker. Non-invasive methods using the combination of buccal swab and urinary epithelial cells should be preferred in first line. To successfully identify carriers of the single mtDNA deletion, it is necessary to test (repeatedly) several tissues of the patients (blood, urine, and buccal swab), since the presence of SLSMD might be tissue and time specific.

## Figures and Tables

**Table 1:** General characteristics of 47 symptomatic patients.

	Total (*n*)	PEO	KSS Spectrum	Pearson Syndrome	Atypical
Patients (male/female)	47 (23/24)	17 (7/10)	20 (11/9)	5 (2/3)	5 (3/2)
Deceased patients	9	0	6	2	1
Age of onset (years)	17.2 ± 10.8	20.5 ± 12	18.2 ± 12.2	0.28 ± 0.4	29.7 ± 26.7
Age of death (years)	31.5 (0.5–66)	0	37 ± 21.3	0.75	57

Atypical, patients with atypical phenotype; KSS, Kearns-Sayre Syndrome Spectrum; *n*, number; PEO, Progressive External Ophthalmoplegia.

**Table 2:** Summary of clinical features of 42 patients.

	Total *n* = 42 (%)	PEO *n* = 17 (%)	KSS Spectrum *n* = 20 (%)	Pearson Syndrome *n* = 5
Ptosis	38 (90)	17 (100)	20 (100)	1
PEO	34 (81)	17 (100)	16 (80)	1
Pigmentary retinopathy	13 (31)	0	12 (60)	1
AV cardiac conduction defects (IIIrd degree)	5 (12)	0	5 (25)	0
Cardiomyopathy	1 (2)	0	1 (5)	0
Myalgia	11 (26)	3 (18)	8 (40)	0
Exercise intolerance	23 (55)	6 (35)	16 (80)	1
Swallowing difficulties	8 (19)	4 (24)	4 (20)	0
Hearing impairment	10 (24)	0	10 (50)	0
Diabetes mellitus	5 (12)	1 (6)	4 (20)	0
Adrenal insufficiency	2 (5)	0	0	2
Renal involvement	2 (5)	0	2 (10)	0
Cerebellar symptomatology	11 (26)	0	10 (50)	1
Cognitive impairment	5 (12)	0	2 (10)	3
Short stature	9 (21)	0	6 (30)	3
Failure to thrive	6 (14)	0	2 (10)	4
Anaemia	6 (14)	0	1 (5)	5

KSS, Kearns-Sayre Syndrome Spectrum; *n*, number; PEO, Progressive External Ophthalmoplegia.

**Table 3:** Clinical features in patients with *atypical* phenotype.

	P1	P2	P3	P4	P5
Female/male	male	male	male	female	female
Age of onset (years)	1.5	17	15	66	49
Age of death/current age (years)	−/28	57	−/40	−/80	−/70
Presenting complaint	Psychomotor delay	Epilepsy	Dysarthria, hypomimia	Hearing impairment	Myalgia
Exercise intolerance	+	−	+	+	+
Myalgia	+	−	−	+	+
Difficulty swallowing	−	−	+	+	−
Myopathy	−	+	+	+	−
Fatigue	+	−	−	−	−
Epilepsy	−	+	−	−	−
Stroke-like episodes	−	+	−	−	−
Ptosis, PEO	−	−	−	−	−
Hearing impairment	−	−	−	+	−
Cognitive decline	+	−	−	−	−
Hypertrophic cardiomyopathy	+	−	−	−	−
Diabetes mellitus (type II)	−	−	−	−	+
Short stature	+	−	−	−	−

+, presence of the symptom; −, absence of the symptom; P, patient; PEO, Progressive External Ophthalmoplegia.
